# Obesity, beverage consumption and sleep patterns in rural African women in relation to advertising of these beverages

**DOI:** 10.1093/inthealth/ihae031

**Published:** 2024-05-11

**Authors:** Merling Phaswana, Zandile June-Rose Mchiza, Sunday Olawale Onagbiye, Philippe Jean-Luc Gradidge

**Affiliations:** Department of Exercise Science and Sports Medicine, Faculty of Health Sciences, University of the Witwatersrand, Johannesburg, South Africa; Non-Communicable Disease Research Unit, South African Medical Research Council, Francie van Zijl Drive, Parowvallei, Tygerberg, Cape Town, South Africa; School of Public Health, University of the Western Cape, Bellville, South Africa; Department of Health and Human Sciences, Frederick Community College, Maryland, USA; Department of Exercise Science and Sports Medicine, Faculty of Health Sciences, University of the Witwatersrand, Johannesburg, South Africa

**Keywords:** obesity, physical activity, sleep pattern, South Africa, sugar-sweetened beverages, women

## Abstract

**Background:**

The burden of obesity-related, non-communicable diseases in South Africa is persistent, with poor and black South African women particularly vulnerable. The purpose of the present study was to determine relationships between obesity, physical activity, sleep patterns and beverage consumption among black South African women in a rural village in the Limpopo province.

**Methods:**

A cross-sectional study was conducted among 200 rural-dwelling African women. Data were collected on beverage consumption, sociodemographic information, sleep patterns and anthropometry using self-reported questionnaires.

**Results:**

The mean body mass index (BMI) was 28.5±7.3 kg/m^2^, with 40% being classified as obese (BMI ≥30 kg/m^2^) and the mean sleep score was 4.68±2.51. Participants with very bad habitual sleeping patterns consumed significantly more sugar-sweetened beverages and alcohol than those with very good sleeping patterns. We also observed that when total coffee with sugar, fruit juice, total sugar-sweetened beverages and weight decreased the number of hours participants slept increased.

**Conclusions:**

The study identified significant associations between body weight, sleep duration and sugar-sweetened beverage consumption among rural black South African women. This underscores a need to address unhealthy lifestyle behaviours to lower incidences of non-communicable diseases in rural-dwelling women.

## Introduction

Obesity and related non-communicable diseases (NCDs) remain a public health concern globally, resulting in increased premature mortality rates in low- and middle-income countries (LMICs).^1^ South Africa has the highest prevalence of obesity in sub-Saharan Africa (SSA), with rates expected to reach 47.7% among women by 2025.^2^ Less than half of the adult South African population has a healthy weight, with black women exhibiting an obesity incidence of 41%.^3^ Sugar-sweetened beverages (SSBs) have also been viewed as a significant contributor to the burden of obesity and NCDs in SSA.^4^ South Africa, like other nations, responded to the strong recommendation of the World Health Organization (WHO) that governments implement taxation policies on alcohol and SSBs to reduce the consumption of unhealthy beverages and promote healthy dietary practices.^5^ However, it is yet to be confirmed if the implementation of the Health Promotion Levy will reduce the consumption of SSBs and combat the obesity pandemic in the country. This taxation policy initiative is part of the key advocacy interventions of the South African government and non-governmental organizations (NGOs) such as the Healthy Living Alliance (HEALA) to encourage healthy food and lifestyle choices to prevent NCDs.^6^ These initiatives are at the core of a drive to address weight gain, dental caries and NCDs that pose a significant threat to public health in South Africa.

Given the persistence of food insecurity and the scarcity of clean water in LMICs, it remains difficult to replace sugary drinks with plain water and nutrient-rich beverages such as dairy products.^7^ This is especially true in rural and peri-urban communities in South Africa, where women face unique challenges that require special attention. Due to affordability, most of these communities are affected by food insecurity and tend to consume energy-dense foods, which increases their vulnerability to obesity.^8^ This is particularly true for women, who often assume the primary role in ensuring household nutrition and well-being. Socio-economically deprived neighbourhoods have easy access to cheap sugary drinks and alcohol.^9^ These beverages are constantly promoted through repetitive, highly persuasive and well-financed marketing and advertising strategies, with a particular emphasis on targeting women and children.^10,11^ The health risks associated with SSB consumption may be accelerated due to limited resources and social support. In underserved areas, physical activity is declining due to socio-economic disparities, inadequate infrastructure and a lack of awareness about the benefits of exercise.^12^ In these circumstances, women often face additional barriers to exercise, including time constraints and safety concerns. These challenges significantly contribute to the rates of obesity in South African women.^12^

Previous studies have shown that excessive consumption of SSBs and alcohol are associated with sleep disturbance, and this is linked to poor food choices and weight gain.^13^ Moreover, sleep problems are correlated with an increased risk of obesity and NCDs such as type 2 diabetes and hypertension.^14^ There is also evidence that insufficient sleep is associated with weight gain.^13^ A recent review using clinical outcomes and clinical trials reported a strong link between the consumption of SSBs and increased weight gain. Therefore this article aims to contribute to the limited data on SSB consumption and sleep patterns among rural-dwelling black South African women as well as the relationships between obesity, physical activity, sleep patterns and beverage consumption.

## Methods

This study was a cross-sectional survey that included a convenient sample of 200 black women from the Tshino Nesengani (Mukondeleli) village in Limpopo province, South Africa. Participants who were pregnant, <18 y of age, non-black and living outside the village were excluded from the study.

### Anthropometric measurements

Body weight (kg) and height (m) were measured using calibrated Seca weighing scales and Seca stadiometers (Seca Instruments, Hamburg, Germany), with participants wearing minimal clothing. These measurements were used to calculate the body mass index (BMI) of the participants expressed as kg/m^2^. BMI was categorized as underweight (<18.5 kg/m^2^), normal weight (18.5–24.9 kg/m^2^), overweight (25–29.9 kg/m^2^) and obese (≥30 kg/m^2^).^15^ The waist circumference (cm) of the participants was measured using a non-stretch tape measure (Seca Instruments, Hamburg, Germany). Waist circumference measurements ≥80 cm indicated a higher risk of developing obesity-related conditions in women.^16^

### Beverage intake questionnaire

The consumption of SSBs, unsweetened beverages (UBs) and alcohol was determined using the 15-item beverage intake questionnaire (BEVQ-15).^17^ This scale has previously been validated and its reliability tested for use in large-scale investigations.^17^ SSBs are beverages that contain added caloric sweeteners such as sucrose, high-fructose corn syrup (HFCS) or fruit juice concentrates, which include but are not limited to soft drinks, fruit drinks, sports drinks, energy and vitamin water drinks, sweetened iced tea and lemonade. Alcoholic beverages, on the other hand, are beverages with an alcohol content (ethanol or ethyl) ≥1%.^17^

### Physical activity

The Global Physical Activity Questionnaire (GPAQ) was used to determine the self-reported total moderate–vigorous physical activity (MVPA) and estimated time sitting. The GPAQ is reliable and has been validated for use in Africa.^18^ GPAQ active were defined as taking part in the following: moderate physical activity for a total of 150 min/week (≥5 d/week) or vigorous physical activity for 60 min/week (≥3 d/week) or 600 metabolic minutes per week (≥5 d MVPA).^18^ Additionally, walking for travel as a domain of light physical activity was determined using the GPAQ.

### Pittsburgh Sleep Questionnaire Index (PSQI)

Sleep pattern was determined using the PSQI.^19^ The PSQI has been validated for use in African populations.^20^ Individual PSQI items include subjective sleep quality, sleep latency, sleep duration, habitual sleep efficiency, sleep disturbances, sleep medication and daytime dysfunction. Each of these items has a range of 0–3, with 0 indicating no difficulty and 3 indicating severe difficulty. The respective sleep components were scored and categorized as very good (coded as 0), fairly good (coded as 1), fairly bad (coded as 2) and very bad (coded as 3).

### Statistical analysis

Statistica version 13.2 (TIBCO Software, Palo Alto, CA, USA) was used for data analysis. Descriptive statistics, cross-tabulations, chi-squared test and analysis of variance were performed. Data are presented as mean±standard deviation (SD), median (interquartile range [IQR]) and percentages. Significance was determined by p-values ≤0.05.

## Results

A total sample of 200 adult women was enrolled to participate in this study. According to Table 1, the mean age, BMI and waist circumference of the participants were 35.7±16.1 y, 28.5±7.3 kg/m^2^ and 87.7±16.8 cm, respectively. Overall, 5% of the study participants were underweight, 25% were overweight and 40% were obese. Additionally, 95% of the participants consumed SSBs, 66% of the participants consumed unsweetened beverages (UBs) and 12% of the participants consumed alcohol. The median SSBs, UBs and alcohol were 295 kcal (IQR 137–598), 36.4 kcal (IQR 0–174) and 0 kcal (IQR 0–0), respectively. Most reported access to television, radio and mobile phones. The total mean PSQI-derived score was 4.68±2.51, with the respective individual items displayed in Table 1. The median of sitting time and MVPA were 4 (IQR 3–5) and 247 (IQR 120–428).

**Table 1.** Participant demographics, anthropometry, beverage consumption, physical activity and sleep patterns

Variables	Values
Age (years), mean±SD	35.7±16.1
Completed high school, n (%)	100 (50)
Unemployed, n (%)	144 (72)
Anthropometry, mean±SD	
BMI (kg/m^2^)	28.5±7.3
Waist (cm)	87.7±16.8
Beverage consumption, median (IQR)	
Total SSBs (kcal)	295 (137–598)
Total unsweetened beverages (kcal)	36.4 (0–174)
Total alcoholic beverages (kcal)	0 (0–0)
Physical activity, median (IQR)	
Sitting time (h/day)	4 (3–5)
MVPA (min/week)	247 (120–428)
Sleep pattern (0–3, where 3 is very bad), mean±SD	
Daytime dysfunction score	0.7±0.7
Sleep duration score	0.2±0.6
Sleep onset latency score	1.4±1.2
Sleep disturbances score	1.1±0.4
Use of sleep medication score	0.4±0.7
Sleep efficiency score	0.34±0.68
Subjective sleep quality score	0.50±0.69
Global PSQI index	4.68±2.51

kcal: kilocalories.

**Figure 1.**
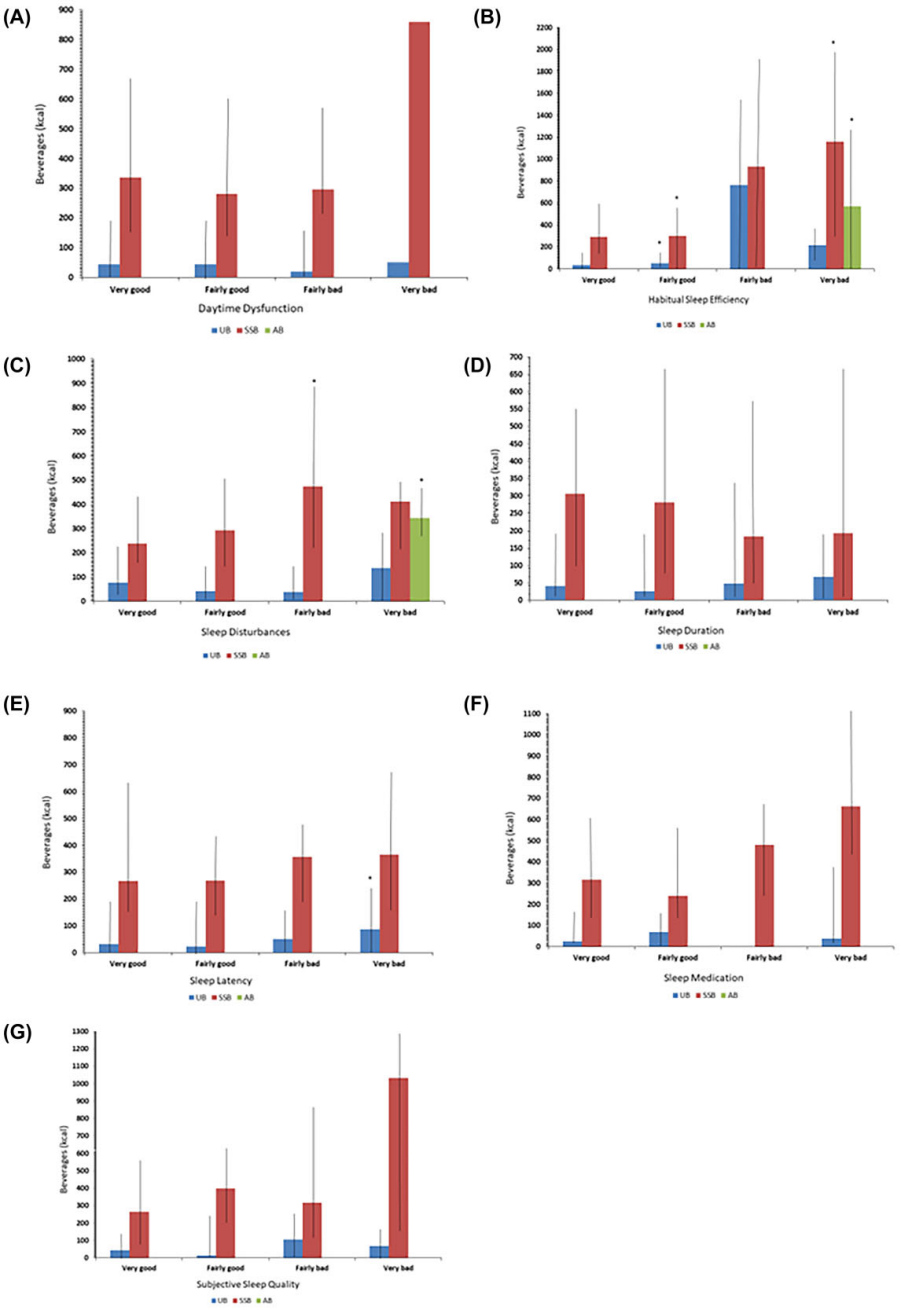
Sleep pattern and beverage consumption.

Figure 1 also shows the chi-squared analysis output suggesting that participants with very bad and fairly good habitual sleep efficiency had a significantly higher consumption of SSBs compared with participants in the very good category. Alcohol consumption was observed in 12% of the participants (Table 1), with those experiencing bad subjective sleep quality consuming more than those in the excellent group. Participants with a very bad habitual sleep efficiency consumed significantly more alcohol than the very good sleepers. Those participants with bad sleep disturbances and daytime dysfunction consumed a greater amount of alcohol than those who did not.

**Figure 2.**
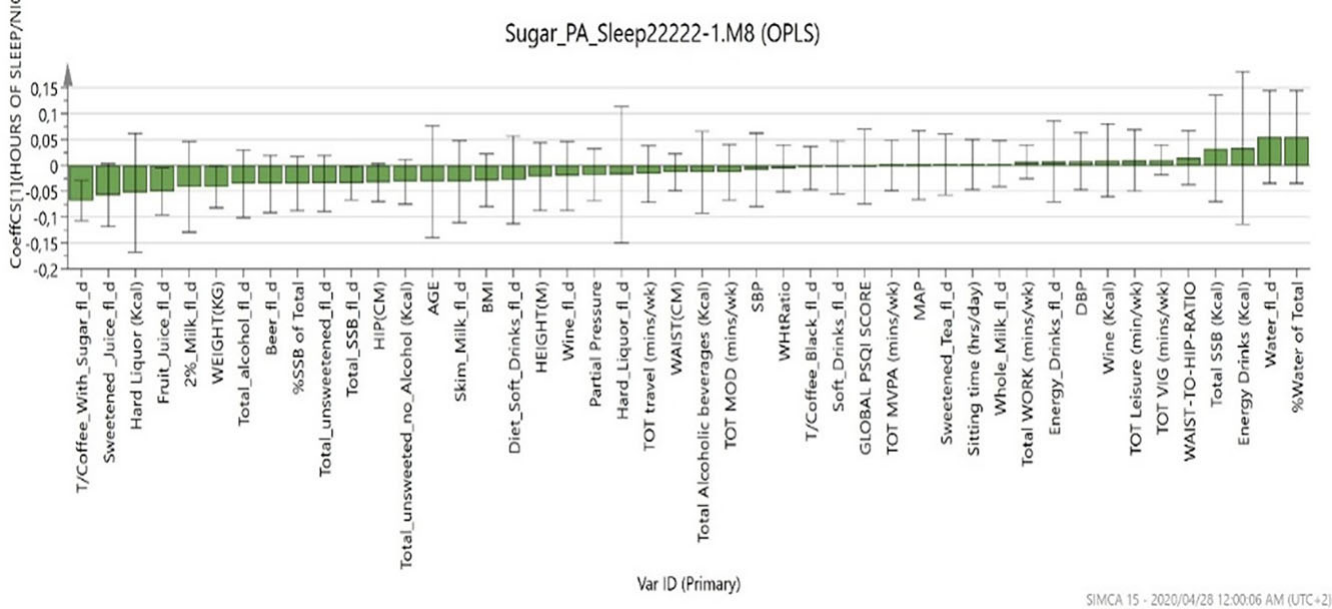
Orthogonal partial least squares analysis.

Figure 2 shows a secondary analysis using regression analysis to examine the association between hours of sleep, anthropometric variables, beverage consumption, physical activity/inactivity and other sleep patterns. The outcomes confirmed that the total volume of coffee with sugar and fruit juice, total SSBs and body weight were the only factors that influenced the total hours of sleep of the participants. For instance, when all these factors decreased, the number of hours of sleep by the participants increased.

## Discussion

This study investigated the relationships between the BMI of black South African women and lifestyle factors such as physical activity, sleep patterns and beverage consumption.

The outcomes of our study suggest that a large majority of the participants (95%) reported consuming more SSBs. These outcomes are corroborated by the outcomes from another longitudinal South African study that reported an exponential growth in the consumption of SSBs in both males (from 26% to 56%) and females (from 33% to 63%) over a 5-y period. However, it is important to highlight that the prevalence recorded in the current study is much higher than the prevalence of 63% observed by Vorster et al.^21^ Beverage advertising directed towards poorer South African communities may contribute to this high prevalence of SSB consumption. Existing South African literature highlight that the majority of both the alcohol and SSB advertisements are shown to vulnerable South African consumers, i.e. women and children, as well as those who live in rural areas.^10,11,22^ Our findings also demonstrate that the consumption of alcohol remains low in this population. This could be attributed to the fact that alcohol is expensive. These findings are consistent with those reported by the World Health Organization (WHO).^23^

The women in the current study are particularly vulnerable given that most of them do not have jobs and in most cases are self-employed or work in domestic and farming activities. As a result, they might be spending more time watching television and engaging in viewing other forms of media and screen time activities. Continued monitoring of advertisements by the Advertising Standards Authority of South Africa is therefore needed to implement strategies such as the HEALA recommendations to reduce SSB and alcohol consumption in the country.^11^

On interrogating the types of SSBs consumed by the study participants we observed that they also consumed homemade beverages and beverages with a high caffeine content (i.e. tea and coffee). Previous evidence has shown that South Africa is a nation that frequently consumes tea and coffee, with the average consumption being about 2 cups/d.^21^ Sugar is usually added to these beverages, with the average amount being 25 g of sugar per cup (approximately 5 heaped teaspoons).^21^ This amount is the maximum amount of daily added sugar recommended by the WHO for adults. Excessive amounts of added sugar in beverages have been implicated in the NCD burden, including obesity and associated sleep disturbance.^24^ It has also been reported that individuals who consume more added sugar (≥10% energy from added sugars) have higher waist circumferences (mean difference 1.07 cm [95% confidence interval {CI} 0.35 to 1.79]) and BMIs (0.43 [95% CI 0.12 to 0.74]) than those who consumed less added sugars.^21^ The participants in our study had a mean waist circumference of 87.7 cm. This is a cause for concern.^25^ However, on exploring the relationships between the BMI of our participants and the amount of SSBs and alcohol they consumed, we could not find any significant associations.

There is existing international evidence that suggests associations between increased BMI and sleep patterns in adults.^13^ The evidence also suggests that sleep patterns are related to demographic characteristics.^24,25^ Al Lawati et al.^26^ showed that an increase in age is associated with sleep disturbance. This assumption seems to corroborate our findings, as we found a significant relationship between age and sleep patterns. Stamatakis et al.^27^ showed that 35% of participants in their study, who were 45–92 y of age, had fewer sleeping hours than their younger counterparts who were <45 y of age (mean 6 h vs 9 h). Stamatakis et al.^27^ explored determinants of short sleep duration among Americans and found that short sleep duration was higher among African Americans and Hispanics, especially those with the lowest household income quintile (odds ratio [OR] 1.62 [95% CI 1.34 to 1.94]) and those with less than a high school education (OR 1.51 [95% CI 1.30 to 1.75]), and among African Americans (OR 1.97 [95% CI 1.68 to 2.30]) when compared with Caucasians. Education and socio-economic status could explain the observed sleep disturbances in our current study population, since the majority belonged to the lowest income quintile, i.e. half of our participants had a high school education with the rest having less than a high school education.

The association between poor sleep and insufficient physical activity has also been acknowledged as an important and growing public health issue.^28^ In fact, Kline^28^ suggested adequate sleep to be a necessity for ideal health and further suggested that poor sleep could predispose individuals to cardiovascular diseases, metabolic dysfunction, psychiatric disorders and premature death, especially when combined with an inactive lifestyle. In the current study, we found that physical activity (especially vigorous activity) was positively associated with hours of sleep despite the association being insignificant. We also demonstrated that every unit increase in physical activity involvement improved sleep quality by an hour.

Our findings also showed that vigorous physical activity was negatively associated with the BMI of our participants. These findings are consistent with other findings obtained when similar research was conducted in another South African female population.^29^ The current evidence also supports the WHO physical activity level recommendations directed at improving adult body weight.

There are limitations to this cross-sectional study that should be recognized. This study included the use of self-reported instruments and participants were limited to a relatively small sample of rural black South African women. Therefore, generalizations to the wider population cannot be made and further investigation is required involving a large sample size and objective measures. The study was conducted before the introduction of the South African health promotion levy (sugar tax). As such, there is a need to monitor the consumption of SSBs and consumer behaviour to determine the effectiveness of the levy, particularly in the study population, as most consumed a high number of SSBs.

## Conclusions

The consumption of SSBs is high, resulting in an elevated risk of obesity-related disease as well as sleep disturbance that further impacts negatively on health. Therefore, it is critical to address the high consumption of SSBs in order to lower the risks of NCDs in South Africa. We recommend for future research a review of South African policy regarding the consumption of SSBs, especially those that also contain caffeine and/or alcohol. This policy requires constant reviewing to evaluate its effectiveness in public health outcomes. Furthermore, the media can be used as a platform to promote healthy food purchases, preferences and obesogenic dietary practices through deliberate policy influence.

## Data Availability

The datasets used in the current study are available from the corresponding author upon reasonable request.
